# Tonsillar metastasis from hepatocellular carcinoma: a case report and literature review

**DOI:** 10.3389/fonc.2026.1901768

**Published:** 2026-09-29

**Authors:** Jianhao Yang, Yujuan Dai, Lusheng Wen, Cuiyu Hu, Guomin Zhang

**Affiliations:** 1 Department of Oncology, The 909th Hospital, School of Medicine, Xiamen University, Zhangzhou, Fujian, China; 2 Department of Pathology, The 909th Hospital, School of Medicine, Xiamen University, Zhangzhou, Fujian, China; 3 Department of Blood Transfusion, The 909th Hospital, School of Medicine, Xiamen University, Zhangzhou, Fujian, China; 4 Department of Otorhinolaryngology, The 909th Hospital, School of Medicine, Xiamen University, Zhangzhou, Fujian, China

**Keywords:** Arginase-1, Batson vertebral venous plexus, hepatocellular carcinoma, immunohistochemistry, tonsillar metastasis, tumor heterogeneity

## Abstract

Tonsillar metastasis from hepatocellular carcinoma (HCC) is extremely rare. We report an 84-year-old man with chronic hepatitis B who presented with a right-lobe HCC and portal vein tumor thrombosis. Three months after diagnosis, intrahepatic metastases were treated with transarterial chemoembolization and lenvatinib. Twenty-three months later, he developed blood-tinged sputum; imaging and laryngoscopy revealed a left tonsillar mass with concurrent pulmonary and hepatic progression. Tonsillectomy yielded an undifferentiated carcinoma lesion. Compared with the primary tumor, the tonsillar metastasis lost hepatocyte specific antigen (Hep Par-1) and Arginase-1 expression, showed a markedly elevated Ki-67 index (80%), but retained Glypican-3 and heat shock protein 70 (HSP70). A diagnosis of metastatic HCC was favored based on integration of clinical history, laboratory findings, and pathological immunohistochemistry. In HCC patients, any unusual head and neck lesion warrants suspicion of metastasis; a multi-marker panel is critical for diagnosis, as loss of Arginase-1 does not rule out HCC.

## Introduction

HCC ranks among the leading causes of cancer-related mortality worldwide, with approximately 906,000 new cases and 830,000 deaths annually (1). Extrahepatic metastasis (EHM) occurs in 5%–15% of HCC patients and portends a dismal prognosis (2), with median overall survival consistently reported between 4.0 and 12.1 months across large cohort studies and systematic reviews (3–5). The most common sites of EHM are the lungs, lymph nodes, bones, and adrenal glands (6). Approximately 1% of all HCC metastases occur in the oral cavity (7). Tonsillar metastasis from hepatocellular carcinoma is even rarer, with only isolated case reports documented in the English literature (8–13).

Herein, we report an extremely rare case of tonsillar metastasis in an elderly male HCC patient who had previously undergone surgical resection, transarterial chemoembolization (TACE), and lenvatinib therapy. Combined with a literature review, we discuss the clinicopathological features, potential metastatic mechanisms (including the Batson vertebral venous plexus hypothesis), and key diagnostic pitfalls of this atypical presentation.

## Case description

An 84-year-old man with a history of chronic hepatitis B had received long-term antiviral therapy with entecavir and achieved sustained virologic suppression. He had no history of alcohol consumption and had a 50-year smoking history of approximately 20 cigarettes per day. There was no family history of malignant tumors, including liver cancer.

In Jan 2023, he was admitted to our hospital due to epigastric distension and discomfort for 1 week. Physical examination revealed a soft abdomen, deep tenderness in the right upper quadrant, positive liver percussion tenderness, negative Murphy’s sign, no palpable abdominal mass, negative shifting dullness, and normal bowel sounds.

Laboratory examinations revealed markedly elevated tumor markers, with alpha-fetoprotein (AFP) of 8851.95 ng/mL (reference range: 0–7.32 ng/mL) and protein induced by vitamin K absence-II (PIVKA-II) of 23361.2 mAU/mL (reference range: 0–40 mAU/mL). Liver function assessment showed alanine transaminase (ALT) 14.3 U/L (reference range: 9.0–50.0 U/L), aspartate transaminase (AST) 182.3 U/L (reference range: 15.0–40.0 U/L), and albumin (ALB) 35.0 g/L (reference range: 40–55 g/L). Total bilirubin was 23.07 μmol/L (reference range: 0–26 μmol/L), and prothrombin time was 13.3 s (reference range: 9.8–12.1 s), slightly prolonged. Hepatitis C antibody was negative. The patient presented with negative hepatitis B e antigen surface antigen (HBsAg, 0.01 IU/mL; reference: 0–0.05 IU/mL), low and non-protective hepatitis B surface antibody (HBsAb, <1.87 mIU/mL; reference: 0–10.00 mIU/mL), (HBeAg, <0.2 PEIU/mL; reference: 0–0.70 PEIU/mL), and positive hepatitis B e antibody (HBeAb, >3.00 IU/mL; reference: 0–0.20 IU/mL) and hepatitis B core antibody (HBcAb, >10.00 IU/mL; reference: 0–0.60 IU/mL). HBV DNA was <20 IU/mL (undetectable). The patient had been on long‑term entecavir therapy with sustained virologic suppression. Abdominal portal‑venous‑phase MRI demonstrated a portal‑vein posterior‑branch filling defect suggestive of primary HCC with tumor thrombosis, without features of cirrhosis or ascites (**Figures 1A, B**). The patient was clinically staged as Barcelona Clinic Liver Cancer (BCLC) stage C, with Child‑Pugh class A hepatic function. After obtaining comprehensive informed consent regarding the risks and benefits of the procedure, the patient underwent right hepatectomy, portal vein tumor thrombus thrombectomy, and cholecystectomy. Postoperative pathological examination confirmed Edmondson–Steiner grade II hepatocellular carcinoma with gross portal vein tumor thrombus.

**Figure 1:**
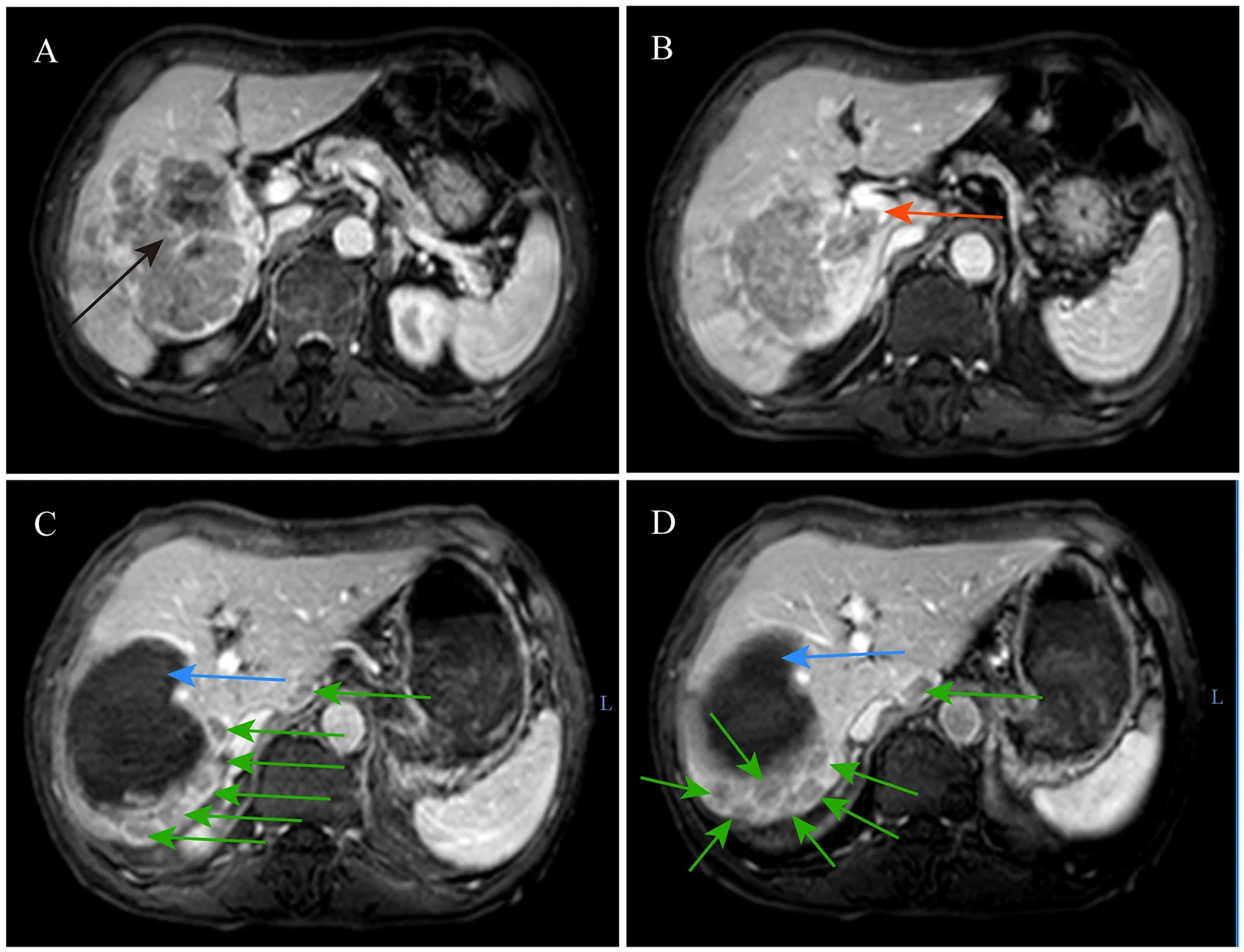
Imaging changes of liver lesions before and after surgery. **(A, B)** Portal venous-phase MRI before surgery showed a large mass in the right hepatic lobe (black arrow) with tumor thrombus in the right portal vein (red arrow). **(C)** Portal venous-phase MRI at 3 months postoperatively showed encapsulated fluid collection at the surgical site (blue arrows) and multiple intrahepatic metastases (green arrows). **(D)** Portal venous-phase MRI at 1 year postoperatively showed stable encapsulated fluid collection, with slight enlargement of multiple intrahepatic metastatic lesions (green arrows) compared with the prior study.

In April 2023, follow‑up liver MRI showed multiple new intrahepatic nodules around the surgical resection site, consistent with metastases (**Figure 1C**). The patient’s BCLC stage C and Child‑Pugh class A remained unchanged. The patient received TACE in April 2023 and again in May 2023. Concurrently, he was initiated on oral lenvatinib at a weight-based dose of 8 mg once daily (actual body weight <60 kg), according to standard clinical practice and CSCO guidelines. One year after the initial surgery (January 2024), imaging showed mild tumor enlargement meeting RECIST criteria for stable disease (**Figure 1D**). Continuation of lenvatinib with closer surveillance was recommended, but the patient and his family declined further follow-up visits. Nevertheless, he presented again to our hospital on December 19, 2024, with blood-tinged sputum for 8 days. Physical examination revealed no erythema or swelling of the right tonsil, but ulceration with a dark necrotic pseudomembrane on the left. Electronic laryngoscopy revealed ulceration of the left tonsil and a dark red mass at the base of the tongue (**Figure 2A**). Oral‑pharyngeal MRI demonstrated soft‑tissue thickening of bilateral tonsils, predominantly on the left, with the largest cross‑section of 2.7 cm × 1.9 cm, suspicious for malignancy (**Figure 2B**). Chest CT confirmed multiple pulmonary metastases (**Figure 2C**), indicating further disease progression. Serum tumor markers were markedly elevated, with AFP >24200 ng/mL and PIVKA-II >30000 mAU/mL.

**Figure 2:**
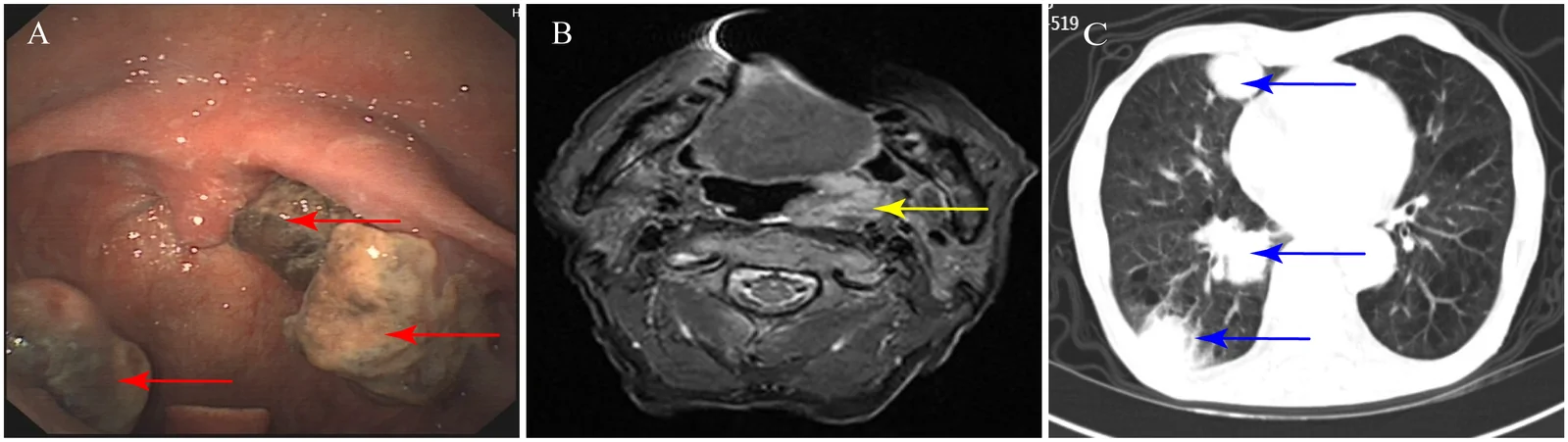
Endoscopic and imaging findings of tonsillar metastasis and systemic metastasis. **(A)** Electronic laryngoscopy showed necrotic neoplasms in the left tonsil and right base of the tongue (red arrows). **(B)** Oropharyngeal MRI revealed soft-tissue mucosal thickening in the left tonsillar region, suggestive of malignancy (yellow arrow). **(C)** Chest CT demonstrated multiple space-occupying lesions in both lungs, consistent with metastatic tumors (blue arrows).

An initial biopsy of the oropharyngeal mass revealed poorly differentiated carcinoma. On December 31, 2024, the patient underwent left tonsillectomy with extended resection of the right pharyngeal lesion. Immunohistochemical profiles of the primary hepatic tumor and the tonsillar metastasis are summarized in **Table 1**. Postoperative histopathology and immunohistochemistry (**Table 1**; **Figures 3A–F**) supported the diagnosis of metastatic HCC (Edmondson‑Steiner grade IV) based on retained GPC3/HSP70/CK8/18 positivity, negative squamous markers, and the clinical history of primary HCC, despite loss of Hep Par‑1 and Arginase‑1 in the metastasis. After a definitive diagnosis of metastatic HCC, the patient declined second-line systemic antitumor therapy. He was discharged on symptomatic supportive care and died of progressive disease 60 days later.

**Table 1:** Comparison of immunohistochemical profiles between hepatic primary lesion and tonsillar metastatic lesion.

Antibody (abbreviation)	Clonality	Source	Result	
			Hepatic primary lesion	Tonsillar metastatic lesion
Hep Par-1	Monoclonal	Maxin Biotech	Focal positive	Negative
Glypican-3	Monoclonal	Maxin Biotech	Positive	Positive
Arginase‑1	Monoclonal	Maxin Biotech	Positive	Negative
HSP70	Monoclonal	Maxin Biotech	Positive	Positive
CK8/18	Monoclonal	Maxin Biotech	Positive	Positive
p40	Monoclonal	Maxin Biotech	Not done	Negative
CK5/6	Monoclonal	Maxin Biotech	Not done	Negative
Ki-67	Monoclonal	Maxin Biotech	50%	80%

Reagents: Ready-to-use formulations supplied by Maxin Biotech Co., Ltd. (Fuzhou, China), no dilution required prior to experimentation.

**Figure 3:**
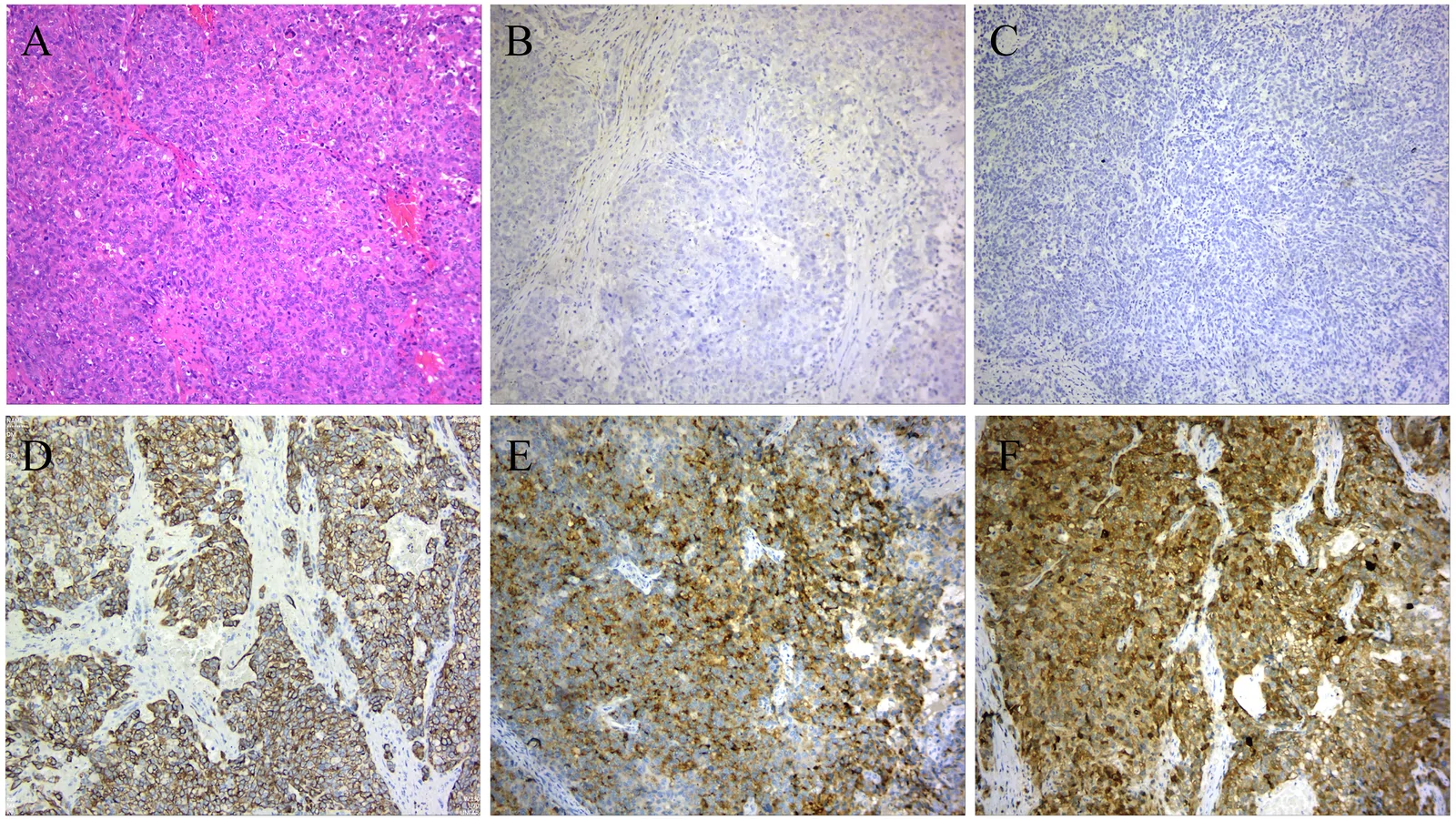
Histopathology and immunohistochemical features of the tonsillar metastatic lesion (original magnification ×100). **(A)** Hematoxylin and eosin staining shows sheets of tumor cells with invasive growth. **(B)** Hep Par-1 is negative. **(C)** Arginase-1 is negative. **(D)** Cytokeratin 8/18 (CK8/18) shows strong positivity. **(E)** Glypican-3 is positive. **(F)** HSP70 shows strong positivity.

## Discussion

We searched PubMed and Web of Science up to December 2025 using the keywords “hepatocellular carcinoma” (or HCC) in combination with “tonsillar metastasis”, “palatine tonsil metastasis”, or “oropharyngeal metastasis”. We included original case reports or case series with sufficient clinicopathological data of histopathologically confirmed HCC metastatic to the palatine tonsil. Articles without full text, those not in English, or describing metastases to adjacent pharyngeal structures without clear tonsillar involvement were excluded. Reference lists were manually screened. This search yielded 6 eligible cases, summarized in **Table 2** alongside our case. The novelty of the present case lies in two aspects: first, we provide a detailed comparison of immunohistochemical (IHC) profiles between the primary hepatic tumor and the tonsillar metastatic lesion, and describe marked phenotypic changes in the metastatic lesion that may be attributable to tumor dedifferentiation or intratumoral-sampling-related bias; Second, the cases summarized in **Table 2** mostly had poor clinical outcomes; however, these observations are limited to the few reported patients and cannot be generalized.

**Table 2:** Metastatic hepatocellular carcinoma involving the tonsil: cases in the current literature.

Author (ref)	Age (y)	sex	Underlying condition	Symptoms/signs	Other metastasis	Therapy	Prognosis (months)
Llanes, F (8)	71	M	HBV&HCV	Lump	None	Tonsillectomy	10 (died)
Endo, Y (9)	58	M	Not available	Bleeding	Lung	Tonsillectomy	NA
Wen, H (10)	66	M	Not available	Lump	None	Tonsillectomy	9 (died)
Karamitsou, C (11)	84	M	HBV	Dysphagia	Rib	None	9.5 (died)
Chalothorn, T (12)	63	M	HCV	Bleeding	Ethmoid sinus, Medial orbit	Radiotherapy	NA
Francis, A (13)	77	M	Chronic liver disease	Lump	Pelvic lymph node	Tonsillectomy Atezo/Bev	30 (alive)
This case	84	M	HBV	Bleeding	Lung	Tonsillectomy	2 (died)

Tonsillar metastases lack pathognomonic clinical manifestations and typically present with sore throat, dysphagia, pharyngeal foreign‑body sensation, bleeding or palpable masses, which are often misdiagnosed as primary tonsillar squamous cell carcinoma or non‑Hodgkin lymphoma. Frances and colleagues described a case where tonsillar enlargement constituted the initial clinical manifestation of underlying HCC, highlighting that tonsillar metastasis can occur prior to detection of the primary hepatic tumor and create substantial diagnostic challenges (13). In our case, the patient presented predominantly with blood-tinged sputum, and physical examination revealed tonsillar ulceration covered with dark necrotic pseudomembrane, further confirming the atypical clinical features of this rare metastatic pattern. Accordingly, all uncharacteristic head‑and‑neck lesions in patients with a known history of HCC should be included in the differential diagnosis of metastatic HCC.

A distinctive clinical feature of our case is tonsillar metastasis occurring after TACE plus lenvatinib treatment, with synchronous radiological progression of pulmonary and intrahepatic lesions. Given the concurrent lung metastases, conventional hematogenous spread represents the preferred explanation for tonsillar metastasis. The Batson vertebral venous plexus—a valveless, bidirectional venous network—has been proposed as an alternative route for HCC cells to bypass pulmonary filtration in patients without detectable pulmonary metastases, and has been invoked to explain isolated head‑and‑neck and spinal metastases (14–17) as well as rare metastatic patterns in prostate and renal‑cell carcinoma (18, 19). However, this hypothesis has not been substantiated by direct evidence across tumor types. Moreover, given the synchronous pulmonary metastases in our patient, invoking this pathway would be a stretch, and the mechanism remains speculative in the present case.

IHC plays an indispensable role in diagnosing metastatic HCC, especially for poorly differentiated or dedifferentiated metastatic lesions that cannot be morphologically distinguished from metastatic squamous cell carcinoma, adenocarcinoma or lymphoma using routine hematoxylin‑eosin staining alone. In this case, the primary HCC was Edmondson–Steiner grade II with focal Hep Par-1 and intact Arginase‑1 immunoreactivity, whereas the tonsillar metastasis corresponded to Edmondson–Steiner grade IV and demonstrated complete absence of both markers, alongside a sharp elevation in the Ki‑67 proliferation index from 50% to 80%. These differences may reflect true biological progression during dissemination, but they may equally be explained by intratumoral heterogeneity or sampling bias in the primary tumor. Loss of hepatocellular markers and the apparent increase in Ki-67 are compatible with tumor dedifferentiation and enhanced aggressiveness (20); however, they are also compatible with undersampling of a heterogeneous primary tumor. Although clonal evolution has been reported in HCC (21), it cannot be inferred here without genomic data. Routine sampling may underestimate intratumoral heterogeneity: Edmondson–Steiner grade is readily underestimated in core biopsies (22), and multiregional analyses detect intratumoral heterogeneity in 87% of HCC cases, most commonly as morphological and immunohistochemical variability (23). Thus, unsampled high-grade or marker-negative regions within the primary tumor cannot be excluded as the source of the observed phenotypic differences. Immunohistochemistry alone cannot distinguish dedifferentiation from sampling-related heterogeneity; multi-region sampling and genomic profiling would be required. These findings should therefore be considered hypothesis-generating.

Arginase‑1 is a sensitive and specific marker of hepatocellular differentiation, with expression correlated with HCC grade (24). Nguyen et al. reported Arginase‑1 sensitivities of 100%, 96.2%, and 85.7% for well‑, moderate‑, and poorly‑differentiated HCC, versus 46.4% for HepPar‑1 in poorly differentiated tumors (25). Arginase‑1 also shows high specificity. In 1,240 specimens, Timek et al. found 100% specificity (no false‑positives in non‑hepatic malignancies), compared with 2.7% for HepPar‑1 and 3.1% for Glypican‑3, supporting a three‑marker panel (Arginase‑1, HepPar‑1, Glypican‑3) (26). Larson et al. confirmed Arginase‑1’s superior specificity and interobserver agreement over HepPar‑1 (27). Complete loss of Arginase‑1 in our oropharyngeal metastasis aligns with its known downregulation in poorly differentiated HCC. Isolated loss of Arginase‑1 or HepPar‑1 does not exclude metastatic HCC; diagnosis requires a panel including Glypican‑3, HSP70, and cytokeratins. Hong et al.’s analysis of 10 patients with oral metastatic HCC (OMHCC) provides valuable practical guidance for clinical management. The median overall survival of OMHCC patients was only 16.9 months, and survival after oral metastasis diagnosis decreased to 13.1 months, indicating that oral metastasis represents end‑stage disease with markedly shortened survival (28). The poor outcome in our patient reflects the advanced stage at diagnosis (BCLC C with portal vein tumor thrombus) and disease progression despite therapy. This aggressive course is consistent with the elevated Ki‑67 in the metastasis (50% to 80%), a well‑established adverse prognostic factor in HCC (29, 30). Palliative surgical intervention is indicated for spontaneous bleeding, intractable pain and dysphagia caused by progressive tumor enlargement. Our patient received palliative tonsillectomy for intractable blood‑tinged sputum, which achieved effective local symptom relief despite continuous systemic tumor progression.

Taken together, clinical management of tonsillar HCC metastasis should adhere to the principle of prioritizing systemic disease control with supplementary palliative local therapy. Local surgical resection or radiotherapy is mainly indicated to relieve bleeding, pain and dysphagia and improve quality of life, rather than achieving curative resection. Currently, no evidence‑based consensus exists regarding adjustments to systemic therapy after resection of rare‑site metastases. Nevertheless, the emergence of distant metastasis indicates tumor resistance to or evasion of current treatment, and individualized therapeutic escalation should be cautiously considered.

Several limitations should be acknowledged. First, as a single‑case report with only six previously documented cases, this study is inherently descriptive. Its findings should be interpreted in the context of this rare observation and not generalized to broader patient populations. Second, the diagnosis of tonsillar metastatic HCC in this case is established through a diagnostic‑of‑exclusion approach, instead of being confirmed by positive hepatocyte‑specific markers. Both Hep Par‑1 and Arginase‑1, two markers highly specific for hepatocellular differentiation, were completely absent in the metastatic lesion. Diagnosis was therefore based on the combined expression of GPC3 and HSP70 (which are not exclusively liver‑specific), negative staining for squamous cell carcinoma markers, together with the consistent clinical and pathological background, which carries inherent diagnostic caveats. Third, we did not perform genomic sequencing. Therefore, we cannot distinguish whether the observed phenotypic discrepancies between primary and metastatic lesions arise from tumor dedifferentiation during metastatic progression or from pre-existing intratumoral heterogeneity within the primary tumor.

## Conclusion

In summary, we report a rare case of tonsillar metastasis from HCC with detailed immunohistochemical profiling of both primary and metastatic lesions. Three observations merit emphasis. First, atypical head and neck metastasis can occur despite TACE-lenvatinib therapy; the Batson plexus offers a plausible but unproven anatomical hypothesis for this unusual route. Second, concurrent loss of Arginase‑1 and elevated Ki‑67 may result from phenotypic dedifferentiation upon metastatic spread or may reflect pre‑existing intratumoral heterogeneity within the primary tumor; both possibilities should be considered when interpreting this finding. Third, negative staining for a single marker does not exclude hepatic origin; multi‑marker panels integrated with clinical history remain essential for accurate diagnosis.

## Data Availability

The original contributions presented in the study are included in the article/**Supplementary material**. Further inquiries can be directed to the corresponding authors.

## Supplementary material

Supplementary Figure 1 Hematoxylin and eosin (H&E) staining and Arginase‑1 immunohistochemistry of the tonsillar metastasis in three distinct areas (original magnification ×100). **(A)** H&E staining showing the superficial portion of the metastatic lesion, with overlying normal stratified squamous epithelium of the palatine tonsil (green arrow) and underlying tumor tissue. **(B)** H&E staining of the central portion of the metastatic lesion. **(C)** H&E staining at the tumor–muscle interface, showing the invasive margin of the metastatic lesion in close proximity to the adjacent muscular layer (red arrow). **(D)** Arginase‑1 immunostaining of the corresponding superficial area, showing complete negativity. **(E)** Arginase‑1 immunostaining of the corresponding central portion, showing complete negativity. **(F)** Arginase‑1 immunostaining of the corresponding tumor–muscle interface, showing complete negativity.
